# Supplementary material for: Quantum walk coherences on a dynamical percolation graph
Source: Sci Rep. 2015 Aug 27;5:13495. doi: 10.1038/srep13495 (PMC4550913; doi:10.1038/srep13495)
Supplement: Supplementary Material [file srep13495-s1.pdf]

Supplementary Material: Quantum walk coherences on a dynamical percolation graph

Fabian Elster,[1, *] Sonja Barkhofen,[1] Thomas Nitsche,[1] Jaroslav Novotný,[2] Aurél Gábris,[2, 3] Igor Jex,[2] and Christine Silberhorn[1]

[1] *Applied Physics, University of Paderborn, Warburger Straße 100, 33098 Paderborn, Germany*
[2] *Department of Physics, Faculty of Nuclear Sciences and Physical Engineering, Czech Technical University in Prague, Břehová 7, 11519 Prague, Czech Republic*
[3] *Department of Theoretical Physics, University of Szeged, Tisza Lajos körút 84, 6720 Szeged, Hungary*

(Dated: April 21, 2015)

I. SUPPLEMENTARY MATERIAL

A. Reduction of the configuration space

A percolation quantum walk (PQW) on the set of configurations $\mathcal{K}$ and another PQW on the restricted set $\mathcal{K}'$ approach the same asymptotic evolution if their asymptotic spectrum and corresponding attractors are the same[1]. Each element $\hat{X}$ of the set of attractors is determined by the set of configurations $\mathcal{K}$ via the equations

$$\left(\hat{S}_\kappa \hat{C}\right) \hat{X} \left(\hat{S}_\kappa \hat{C}\right)^\dagger = \lambda \hat{X}, \qquad \forall \kappa \in \mathcal{K}. \tag{1}$$

These equations can be expanded into so-called shift conditions

$$\hat{S}_\kappa^\dagger \hat{X} \hat{S}_\kappa = \hat{S}_\iota^\dagger \hat{X} \hat{S}_\iota, \qquad \forall \kappa, \iota \in \mathcal{K} \tag{2}$$

responsible for the attractor structure only, and a coin condition

$$\hat{C} \hat{X} \hat{C}^\dagger = \lambda \hat{S}_\kappa \hat{X} \hat{S}_\kappa^\dagger, \tag{3}$$

providing possible candidates for the asymptotic spectrum and related coin-block structure of the attractor[2,3]. To make sure that the restricted set $\mathcal{K}'$ establishes the equivalent set of coin and shift conditions as the full set $\mathcal{K}$, the set $\mathcal{K}'$ has to satisfy the following two sufficient conditions. First, according to the shift conditions, for any pair of graph links e_1, e_2 there is a configuration $\kappa \in \mathcal{K}'$ which contains the link e_1 and the link e_2 is missing, and a configuration $\kappa \in \mathcal{K}'$ which contains the link e_2 but not the link e_1. Second, according to the coin conditions, there is a configuration $\kappa \in \mathcal{K}'$ which includes an isolated vertex. The minimal set of configurations $\mathcal{K}'$ which satisfies both of these conditions is the set which contains all configurations with only one link present.

B. Measurements for vertically polarised input state

All measurements have been carried out both for a horizontal and vertical input polarisation states. In the main text only the data corresponding to horizontal input is presented, since the vertical input shows essentially identical features. In this section we present the results for vertical input for the sake of completeness. The results demonstrate the same fidelity regarding the realization of the dynamically changing graph indicated by the low, less than 2.8 % on average, residual populations outside the three central positions. After averaging over six steps, we obtain 94.1 % for the similarity of the experimental data and the ideal theory, and 94.7 % for the case of the realistic theory data adapted for systematic errors. Extended data figure. 1, the vertical input polarization counterpart of fig. 3 from the main text, serves to illustrate the good agreement with the presented models.

---

[1] Novotný, J., Alber, G. & Jex, I. Asymptotic evolution of random unitary operations. *Central European Journal of Physics* **8**, 1001–1014 (2010). URL `http://dx.doi.org/10.2478/s11534-010-0018-8`.

* fabian.elster@uni-paderborn.de

[2] Kollár, B., Kiss, T., Novotný, J. & Jex, I. Asymptotic dynamics of coined quantum walks on percolation graphs. *Physical Review Letters* **108**, 230505 (2012). URL `http://link.aps.org/doi/10.1103/PhysRevLett.108.230505`.

[3] Kollár, B., Novotný, J., Kiss, T. & Jex, I. Percolation induced effects in two-dimensional coined quantum walks: analytic asymptotic solutions. *New Journal of Physics* **16**, 023002 (2014). URL `http://iopscience.iop.org/1367-2630/16/2/023002`.

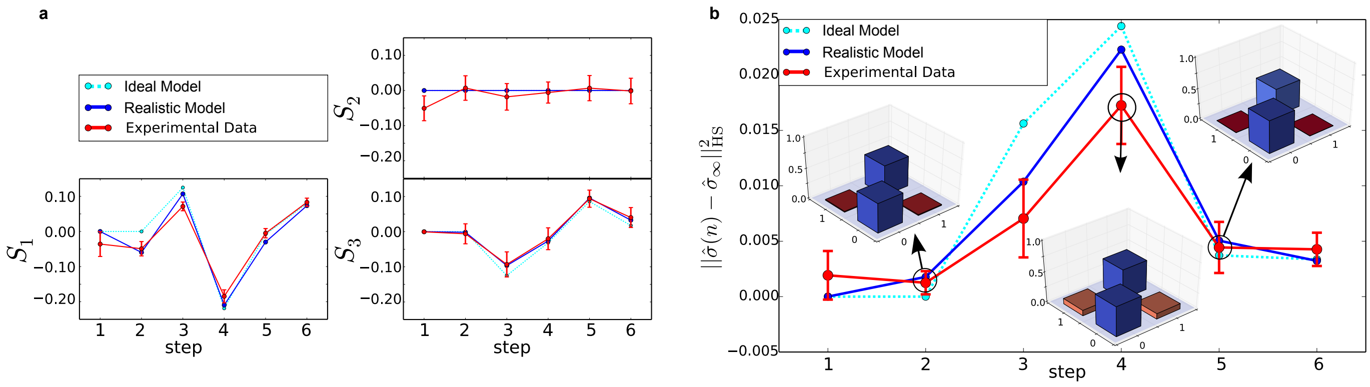

FIG. 1. **Extended data figure 1: Coherence Measurements for vertically polarized input.** **a**: Values of the Stokes parameters of the reduced state $\hat{\sigma}(n)$ of the coin: observed (solid red lines), ideal model (dashed lines), and realistic model (blue, solid lines). **b**: Hilbert–Schmidt distance $||\hat{\sigma}(n) - \hat{\sigma}_\infty||^2_{\mathrm{HS}} \equiv \mathrm{Tr}\left\{(\hat{\sigma}(n) - \hat{\sigma}_\infty)^2\right\}$ between $\hat{\sigma}(n)$ and $\hat{\sigma}_\infty$, the maximally mixed, asymptotic state: observed data (red), ideal model (turquoise), and realistic model (blue). The insets show the experimental density matrix for the three chosen steps. Statistical errors are smaller than the symbol size. The depicted error bars are calculated using a numerical simulation of all relevant systematic errors and are discussed in detail in the Methods.
